# Comparative Meta-Analysis of Left Ventricular Mechanics in Takotsubo Syndrome and Anterior STEMI Due to Left Anterior Descending Artery Occlusion

**DOI:** 10.3390/jcm14248748

**Published:** 2025-12-10

**Authors:** Andrea Sonaglioni, Gian Luigi Nicolosi, Michele Lombardo, Massimo Baravelli, Paola Muti

**Affiliations:** 1 Division of Cardiology, IRCCS MultiMedica, 20123 Milan, Italy; michele.lombardo@multimedica.it (M.L.); massimo.baravelli@multimedica.it (M.B.); 2 Division of Cardiology, Policlinico San Giorgio, 33170 Pordenone, Italy; gianluigi.nicolosi@gmail.com; 3 Department of Biomedical, Surgical and Dental Sciences, University of Milan, 20122 Milan, Italy; pmuti26@gmail.com; 4 IRCCS MultiMedica, 20138 Milan, Italy

**Keywords:** takotsubo syndrome, anterior STEMI, differential diagnosis, speckle tracking echocardiography, meta-analysis

## Abstract

**Background:** Takotsubo syndrome (TTS) often mimics anterior ST-elevation myocardial infarction (STEMI) caused by left anterior descending (LAD) occlusion, yet the two entities differ fundamentally in pathophysiology and mechanical behavior. Two-dimensional speckle-tracking echocardiography (2D-STE) enables detailed assessment of left ventricular (LV) deformation beyond conventional ejection fraction (LVEF). This meta-analysis compared global and regional LV strain patterns in TTS versus LAD-related anterior STEMI during the acute phase. **Methods:** A systematic search of PubMed, Embase, and Scopus through October 2025 identified observational case–control studies directly comparing TTS and angiographically confirmed anterior STEMI, with LV mechanics assessed by 2D-STE. Random-effects models were used to pool standardized mean differences (SMDs) for LVEF; global longitudinal strain (GLS); apical, mid-ventricular, and basal longitudinal strain (ALS, MLS, BLS); and global radial strain (GRS). Heterogeneity (I^2^), publication bias (funnel plots, Egger’s test), meta-regression, and leave-one-out sensitivity analyses were performed. **Results:** Six studies comprising 221 TTS and 290 anterior STEMI patients met the inclusion criteria. TTS patients were older, predominantly female, and had fewer metabolic risk factors, while LV size was comparable. LVEF was significantly lower in TTS (SMD −1.149; 95% CI −2.20 to −0.10; *p* = 0.032), with stable findings across sensitivity analyses and no evidence of publication bias. GLS, ALS, MLS, and BLS showed only a non-significant trend toward greater impairment in TTS, and these comparisons were limited by marked inter-study heterogeneity. In contrast, GRS was significantly and consistently more reduced in TTS (SMD −1.284; 95% CI −1.59 to −0.98; *p* < 0.001), indicating more profound global radial dysfunction. Meta-regression showed no significant influence of demographic factors or vendor-specific software on LVEF or GLS differences. **Conclusions:** Compared with LAD-related anterior STEMI, TTS is associated with more severely depressed LVEF and markedly impaired radial strain, while longitudinal strain differences remain inconclusive and suggest only a potential trend toward greater dysfunction, reflecting the limited and heterogeneous evidence. These findings are consistent with diffuse, stress-induced myocardial stunning in TTS and suggest that 2D-STE may aid differentiation between stress cardiomyopathy and ischemic infarction in the acute setting, although longitudinal strain parameters should be interpreted cautiously and regarded as hypothesis-generating.

## 1. Introduction

Takotsubo syndrome (TTS), also known as stress-induced cardiomyopathy, is an acute, transient disorder of left ventricular (LV) function characterized by regional wall motion abnormalities extending beyond a single coronary artery territory, in the absence of obstructive coronary artery disease [1]. It has been increasingly recognized over the past three decades as a distinct clinical entity predominantly affecting postmenopausal women and typically triggered by emotional or physical stress [2].

Large contemporary registries have confirmed that TTS accounts for a non-negligible proportion of patients initially presenting with suspected acute coronary syndrome, with a marked female predominance and a wide spectrum of clinical triggers and outcomes [3,4]. Comprehensive expert panel reviews have summarized the evolving concepts in epidemiology, diagnostic criteria, and management, highlighting TTS as a heterogeneous syndrome rather than a single disease entity [5].

Clinically, TTS often mimics an acute anterior ST-elevation myocardial infarction (STEMI), presenting with chest pain, ST-segment elevation, and elevation of cardiac biomarkers. This phenotypic overlap represents a major diagnostic challenge in the emergency setting, where rapid triage frequently leads to the assumption of STEMI and thus to urgent coronary angiography. Misdiagnosis of TTS as STEMI may result in unnecessary catheterization and delays in appropriate management, emphasizing the need for improved early differentiation between the two entities [2,6]. Despite similar initial presentations, the underlying pathophysiology differs substantially. TTS is thought to represent a form of myocardial stunning related to catecholamine excess and stress-related neurohumoral activation, whereas anterior STEMI is caused by prolonged ischemia due to an occlusive thrombus in the left anterior descending (LAD) artery [7,8].

Several mechanistic studies and reviews have proposed that TTS involves complex interactions between catecholamine toxicity, microvascular dysfunction, and myocardial energy metabolism, leading to transient but sometimes extensive LV dysfunction [9,10]. In contrast, LAD-related anterior STEMI results in irreversible necrosis within a more clearly demarcated perfusion territory, followed by scar formation and chronic remodeling.

Advances in echocardiographic imaging, particularly two-dimensional (2D) speckle-tracking strain analysis, have enabled a more detailed assessment of LV systolic mechanics beyond conventional left ventricular ejection fraction (LVEF). Global longitudinal strain (GLS) has emerged as a sensitive marker of systolic function across a range of cardiac conditions, including acute coronary syndromes and chronic ischemic heart disease [11,12].

In the context of STEMI, speckle-tracking–derived longitudinal strain has been shown to predict infarct size, microvascular obstruction and subsequent LV remodeling, particularly in first anterior infarctions [13,14].

In TTS, strain imaging offers unique insights into the pattern and extent of myocardial dysfunction. Recent literature and expert reviews describe a characteristic apical-to-basal gradient of longitudinal deformation, with severely impaired apical strain and relatively preserved or even hypercontractile basal segments, as well as potential differences in recovery patterns compared with ischemic injury [15,16,17].

Despite the increasing use of strain imaging in both conditions, its specific value in differentiating TTS from anterior STEMI in the acute phase—a clinically relevant diagnostic dilemma—remains uncertain. Improving early discrimination between these entities using strain parameters could, in principle, reduce unnecessary invasive procedures and guide more appropriate management pathways.

Nevertheless, individual comparative studies are limited by small sample sizes, heterogeneous imaging protocols, and variability in strain software, leading to inconsistent estimates of global and regional LV dysfunction. The extent to which longitudinal and radial strain patterns differ between TTS and LAD-related myocardial infarction in the acute setting therefore remains incompletely defined.

Therefore, this meta-analysis aims to address these knowledge gaps by synthesizing available comparative evidence on LV mechanics—particularly GLS, regional longitudinal strain (apical, mid, basal), and LVEF—in patients with TTS versus those with anterior STEMI due to LAD occlusion.

## 2. Materials and Methods

This systematic review and meta-analysis followed the Preferred Reporting Items for Systematic Reviews and Meta-Analyses (PRISMA) recommendations [18] (Supplementary Materials S1). The study protocol was registered in advance with the INPLASY database (ID: INPLASY2025110021) on 9 November 2025 (Supplementary Materials S2).

### 2.1. Search Strategy

Two independent reviewers (A.S. and M.B.) systematically searched PubMed, Embase, and Scopus through October 2025 for all comparative studies evaluating LV function and mechanics by two-dimensional speckle-tracking echocardiography (2D-STE) in patients with TTS and anterior STEMI due to LAD occlusion. The following keywords and Boolean operators were used: “Takotsubo cardiomyopathy” OR “stress cardiomyopathy” OR “broken heart syndrome” OR “Takotsubo syndrome” AND “ST-elevation myocardial infarction” OR “anterior STEMI” OR “LAD occlusion” AND “echocardiography” OR “speckle tracking echocardiography” OR “strain imaging” OR “left ventricular strain” OR “global longitudinal strain” OR “LV mechanics.” No language or temporal restrictions were applied. Reference lists of included papers and relevant reviews were manually searched to identify additional eligible studies. Disagreements between reviewers were resolved by discussion or, when necessary, by consulting a third investigator.

### 2.2. Eligibility Criteria

Studies were included if they met the following criteria: (1) case–control design directly comparing patients with TTS and anterior STEMI due to LAD occlusion; (2) quantitative assessment of LV function using 2D-STE; and (3) available data for at least one of the following parameters in both groups: GLS, regional longitudinal strain (apical, mid-ventricular, or basal), or LVEF. Exclusion criteria were: (1) studies including mixed cardiomyopathies or other causes of transient LV dysfunction (e.g., myocarditis or sepsis-induced cardiomyopathy); (2) absence of a comparison group with angiographically confirmed LAD occlusion; (3) lack of quantitative LV strain measurements; (4) imaging methods other than echocardiography (e.g., cardiac magnetic resonance imaging strain only); and (5) conference abstracts, editorials, case reports, or narrative reviews without original data.

### 2.3. Study Selection and Data Extraction

Two reviewers (A.S. and M.B.) independently screened all titles and abstracts, followed by a full-text assessment of potentially eligible studies. Data were extracted from each study using a standardized collection form that included the following: (1) study characteristics (first author, year of publication, country, and study design); (2) population details (sample size, mean age, and proportion of female participants); (3) imaging protocol (vendor and software used for STE analysis, and timing of echocardiographic assessment—acute phase, recovery, or both); (4) conventional transthoracic echocardiography parameters (chamber dimensions and indices of LV systolic and diastolic function); (5) STE-derived LV functional parameters [GLS, apical longitudinal strain (ALS), mid-ventricular longitudinal strain (MLS), and basal longitudinal strain (BLS), and global radial strain (GRS) when available]; and (6) summary statistics (mean ± SD or median and interquartile range, together with reported *p*-values or confidence intervals). When results were available only in graphical form, numerical data were extracted using WebPlotDigitizer (version 4.6), a validated digital measurement tool. Any discrepancies between reviewers were resolved through discussion and consensus.

### 2.4. Risk of Bias Assessment

The methodological quality and risk of bias were independently evaluated by two reviewers (A.S. and G.L.N.) using the National Institutes of Health (NIH) Quality Assessment Tool for Case-Control Studies [19]. Each study was classified as “good,” “fair,” or “poor” according to the NIH scoring criteria. Inter-rater reliability was assessed using Cohen’s kappa coefficient (κ), and any discrepancies were resolved through discussion and consensus.

### 2.5. Statistical Analysis

Because individual patient data (IPD) were not available for the included studies, it was not possible to formally assess the normality of continuous variables using tests such as Shapiro–Wilk across all datasets. Instead, the distributional characteristics were inferred from the type of summary statistics reported in the primary studies. As most studies presented continuous variables as medians with interquartile ranges (IQRs), the data were treated as potentially non-normally distributed. For this reason, descriptive values extracted directly from each study were retained in their original non-parametric form, and no transformation to mean or standard deviation was applied for descriptive reporting.

For the meta-analytic pooling of echocardiographic parameters—including GLS, ALS, MLS, BLS, GRS, and LVEF—standardized mean differences (SMDs) were calculated using established methods to estimate means and standard deviations from medians and IQRs (Luo et al. [20], Wan et al. [21]). These conversions rely on parametric assumptions and therefore introduce an additional degree of uncertainty, particularly when underlying distributions may deviate from normality. The converted values were used exclusively for the quantitative meta-analysis and not for descriptive summaries.

According to standard speckle-tracking echocardiography conventions, more negative GLS values indicate greater myocardial deformation (i.e., better systolic function), whereas less negative values correspond to reduced deformation. For the purpose of graphical comparison in the forest plot, GLS magnitudes were converted to positive values so that lower values represent worse deformation. Global radial strain, in contrast, is inherently expressed as a positive value.

A random-effects model (DerSimonian–Lairdmethod) was selected a priori to account for anticipated inter-study heterogeneity. Between-study heterogeneity was quantified with the I^2^ statistic, with thresholds of 25%, 50%, and 75% denoting low, moderate, and high heterogeneity, respectively.

Publication bias was assessed visually using Begg’s funnel plots and statistically using Egger’s regression test.

Meta-regression models examined whether cardiovascular risk factors such as hypertension, diabetes, or age contributed to variability in LVEF effect sizes. Additionally, meta-regression analyses were performed to evaluate whether demographic or methodological factors—including age, sex distribution, and STE software vendor—modulated GLS outcomes.

Sensitivity analyses were conducted by sequentially excluding individual studies (leave-one-out approach) to test the robustness of pooled estimates.

All analyses were performed using Comprehensive Meta-Analysis software (version 3.0, Biostat, Englewood, NJ, USA) and IBM SPSS Statistics version 29.0 (Armonk, NY, USA). A two-tailed *p*-value < 0.05 was considered statistically significant.

## 3. Results

### 3.1. Study Selection

**Figure 1:**
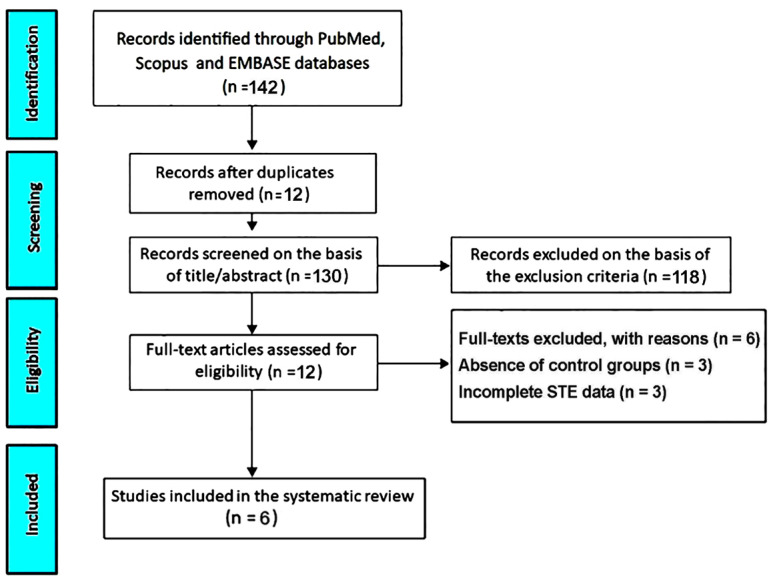
PRISMA flow diagram of study selection for the systematic review.

The initial search yielded 142 records. Twelve studies (8.5%) were removed as duplicates. A further 118 studies (83.1%) were excluded based on the predefined exclusion criteria. The remaining 12 studies (8.5%) were assessed for eligibility. Of these, 3 studies (2.1%) were excluded due to the absence of control groups and 3 (2.1%) due to incomplete STE data. Ultimately, 6 studies (4.2%) [22,23,24,25,26,27] were included in this systematic review and meta-analysis (Figure 1).

### 3.2. Clinical Findings

The included studies were published between 2009 and 2025. They were conducted in South Korea, Germany, the United States, and Sweden, and enrolled a total of 221 patients with TTS and 290 patients with anterior STEMI due to LAD occlusion. All studies assessed LV function using 2D-STE during the acute phase. Diagnostic criteria for TTS were consistent with the Mayo Clinic or European Society of Cardiology (ESC) definitions, while anterior STEMI was confirmed by angiographically proven LAD occlusion treated with percutaneous coronary intervention (PCI). All included studies evaluated only apical variants of TTS; no mid-ventricular, basal, or focal variants were enrolled. Therefore, strain findings reported across the selected literature strictly referred to classical apical ballooning presentations. The timing of echocardiographic assessment varied between studies, ranging from within 24 h to 7 days after symptom onset. Different software packages—GE EchoPAC, TomTec, and Philips QLAB—were used for strain analysis contributing to methodological heterogeneity across studies (Table 1).

The clinical characteristics of TTS and anterior STEMI groups assessed by the included studies are summarized in Table 2.

Across studies, TTS patients were significantly older than those with anterior STEMI (median 68.8 [62.9–73] vs. 63.1 [58–66.9] years, *p* < 0.001) and were predominantly female (91.4% vs. 49.2%, *p* < 0.001). Cardiovascular risk factors were less prevalent in the TTS cohort, including diabetes mellitus (10.9% vs. 20.8%, *p* = 0.004) and hypercholesterolemia (21.8% vs. 41.4%, *p* < 0.001), while hypertension was more frequent in TTS (52.8% vs. 43.3%, *p* = 0.04). The prevalence of active smoking was significantly lower among TTS patients (18.0% vs. 29.9%, *p* = 0.003).

From a hemodynamic perspective, TTS patients exhibited slightly higher heart rates (87.8 [82–94.3] vs. 83.1 [79.9–89.6] bpm, *p* = 0.002) and lower systolic blood pressure (120.4 [98–132.3] vs. 129.1 [119–136.1] mmHg, *p* = 0.003) on admission. ST-segment elevation was observed in approximately half of TTS patients (48%) compared with almost all STEMI cases (98%, *p* < 0.001). Consistent with their distinct pathophysiology, peak troponin I levels were dramatically lower in TTS than in STEMI (2.9 [2.3–3.3] vs. 62.2 [29–93.7] µg/L, *p* < 0.001).

In the TTS population, a physical stressor (41.4%) or an emotional stressor (36.8%) was identified as the most frequent precipitating factor, whereas ischemic chest pain remained the predominant presentation among STEMI patients.

Only two studies reported longitudinal imaging data. Park et al. [22] performed a single follow-up echocardiogram at a mean of 6.7 weeks after the acute event (range 4–16 weeks), documenting near-complete recovery of systolic function in TTS. In contrast, Poller et al. [27] conducted serial strain assessments at days 0, 1, 2, 3, 7, 14, and 30, showing progressive improvement in both groups but more heterogeneous and incomplete recovery among anterior STEMI patients, who frequently exhibited persistent regional wall-motion abnormalities at 30 days.

### 3.3. Conventional Echocardiographic Findings

Across the six studies, conventional transthoracic echocardiography performed during the acute phase revealed consistent differences in LV systolic and diastolic function between patients with TTS and those with anterior STEMI due to LAD occlusion (Table 3).

In line with the pooled results, LVEF was significantly lower in TTS (median 34.2% [25.4–39.7]) compared with anterior STEMI (41.6% [33.1–48.7]; *p* = 0.03), reflecting a more severe, though transient, global systolic dysfunction in the absence of permanent coronary obstruction.

LV structural dimensions were comparable between the two groups. LV end-diastolic diameter (LV-EDD) and end-systolic diameter (LV-ESD) did not differ significantly (46.8 [45–49.1] vs. 47.1 [45–50.7] mm, *p* = 0.44; and 33.3 [29–35.6] vs. 34.2 [32–36.3] mm, *p* = 0.29, respectively), nor did LV end-diastolic and end-systolic volume indices (LV-EDVi 62.5 [49.5–80] vs. 62.7 [45.2–90] mL/m^2^, *p* = 0.96; LV-ESVi 36.9 [29.6–43] vs. 35.2 [26.2–51] mL/m^2^, *p* = 0.61). These findings indicate that the reduction in LVEF in TTS was not driven by increased chamber size but by diffuse, reversible myocardial stunning.

Conversely, the interventricular septal (IVS) thickness was slightly lower in TTS (9.4 [8.9–10.2] vs. 10.0 [9.5–10.3] mm; *p* = 0.03), consistent with a lower myocardial mass and the typically non-hypertrophic LV phenotype observed in stress cardiomyopathy.

Indices of diastolic function demonstrated similar filling pressures between the groups. The E/e′ ratio was nearly identical (12.2 [11.4–13.0] vs. 12.3 [10.3–16.3]; *p* = 0.88), whereas the E/A ratio tended to be higher in TTS (1.00 [0.7–1.3] vs. 1.15 [1.0–1.3]; *p* = 0.07), suggesting a pattern of mild delayed relaxation rather than restrictive filling. The left atrial volume index (LAVi) was comparable or slightly lower in TTS (30.8 [30.7–30.9] vs. 31.4 [31.2–31.7] mL/m^2^; *p* = 0.05), further supporting less chronic diastolic load.

Right ventricular parameters showed distinct differences. Tricuspid annular plane systolic excursion (TAPSE) was significantly lower in TTS (15 vs. 20 mm; *p* = 0.004), indicating greater right ventricular systolic impairment, a well-recognized feature of Takotsubo cardiomyopathy involving biventricular dysfunction. Conversely, right ventricular inflow tract (RVIT) dimensions were similar between groups (37 vs. 36 mm; *p* = 0.41).

Taken together, these findings depicted a characteristic echocardiographic pattern in TTS: more profound, transient global systolic impairment with relatively preserved LV volumes and diastolic function, accompanied by biventricular involvement and reduced TAPSE. This pattern contrasts with anterior STEMI, in which systolic dysfunction was regionally confined to the LAD territory and was typically accompanied by greater LV hypertrophy and higher filling pressures.

### 3.4. Myocardial Strain Parameters

Quantitative strain analysis by 2D-STE demonstrated significant differences in myocardial deformation between TTS and anterior STEMI during the acute phase (Table 4).

Across the six studies included, LV-GLS was significantly reduced in TTS compared with anterior STEMI (median 9.1 [3.5–13] vs. 10.5 [6.3–13]%, *p* = 0.01), indicating more extensive impairment of longitudinal systolic function. Similarly, apical longitudinal strain (LV-ALS) was markedly lower in TTS (4.4 [0.5–9.8] vs. 6.4 [3.4–10]%, *p* = 0.001), reflecting the characteristic apical ballooning and regional dysfunction beyond the LAD territory.

At the mid-ventricular level, LV-MLS was also significantly lower in TTS (9.9 [5.8–13.4] vs. 11.6 [5.7–17.4]%, *p* = 0.006), whereas LV-BLS showed a smaller but still significant difference (14.2 [11.4–18.1] vs. 14.2 [7.3–17.4]%, *p* = 0.03). This gradient pattern—more pronounced impairment toward the apex—illustrated the classical apical-to-basal recovery profile of TTS.

Conversely, global radial strain (LV-GRS) was substantially lower in TTS compared with anterior STEMI (15.7 [13.9–17.5] vs. 26.4 [23.8–29.1]%, *p* < 0.001), suggesting that radial myocardial thickening was globally impaired in TTS, consistent with the diffuse nature of myocardial stunning.

Overall, TTS showed significantly lower GRS and a trend toward more impaired longitudinal strain (GLS, ALS, MLS), with a clear apical-to-basal gradient, whereas anterior STEMI demonstrated more localized abnormalities.

### 3.5. Effect of Takotsubo Syndrome Versus Anterior STEMI on LVEF

Six studies [22,23,24,25,26,27] comparing LVEF between TTS and anterior STEMI were included in the quantitative analysis. Given the high degree of heterogeneity across studies (*Q* = 121.4, *p* < 0.001; I^2^ = 95.9%; τ^2^ = 1.63), a random-effects model was applied.

**Figure 2:**
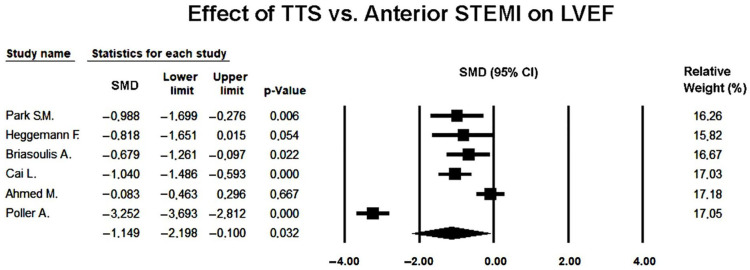
Forest plot showing the effect of TTS vs. anterior STEMI on LVEF [22,23,24,25,26,27]. LVEF, left ventricular ejection fraction; STEMI, ST-elevation myocardial infarction; TTS, takotsubo syndrome.

The pooled SMD in LVEF was −1.149 (95% CI −2.198 to −0.100; *p* = 0.032), indicating that patients with TTS had significantly lower ejection fraction than those with anterior STEMI during the acute phase (Figure 2).

This finding reflected a greater degree of global systolic impairment in TTS, consistent with the diffuse and transient nature of myocardial dysfunction in stress-induced cardiomyopathy.

Despite the consistent direction of effect across studies, substantial heterogeneity persisted, likely reflecting differences in imaging timing, patient selection, and echocardiographic quantification techniques.

**Figure 3:**
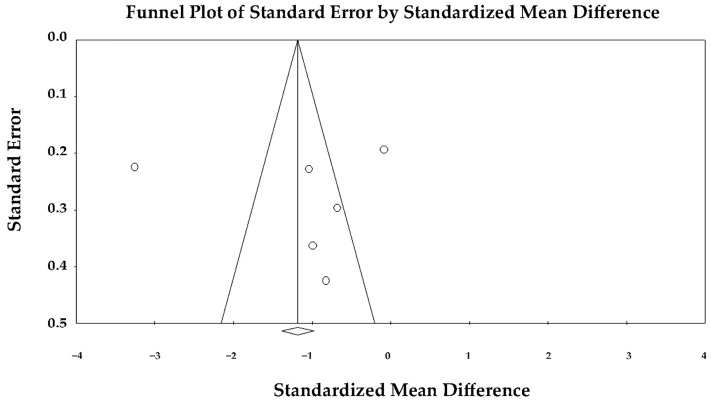
Begg’s funnel plot used to assess publication bias in studies examining the impact of TTS compared with anterior STEMI on LVEF. LVEF, left ventricular ejection fraction; STEMI, ST-elevation myocardial infarction; TTS, takotsubo syndrome.

The funnel plot appeared symmetric (Figure 3), and Egger’s regression test did not reveal any evidence of publication bias (intercept = 0.30 ± 8.73; *t*(4) = 0.03; *p* = 0.97). Therefore, the observed heterogeneity was unlikely to be explained by selective reporting.

Meta-regression analysis did not identify age, prevalence of hypertension, or prevalence of diabetes as significant moderators of the LVEF effect size (Table 5).

None of these covariates showed a statistically significant association with the difference in LVEF between TTS and anterior STEMI (Mean age: coefficient −0.086, *p* = 0.65; %Hypertension: coefficient 0.055, *p* = 0.29; %Diabetes: coefficient 0.133, *p* = 0.27). The overall test of moderators was not significant (Q = 1.66, df = 3, *p* = 0.64), indicating that these clinical factors did not explain the substantial between-study heterogeneity. The model accounted for approximately 11% of the between-study variance (R^2^ analog = 0.11), suggesting that other unmeasured study-level factors likely contributed to variability in effect sizes.

In summary, the random-effects meta-analysis demonstrated that LVEF was significantly lower in TTS compared with anterior STEMI, indicating more pronounced acute systolic dysfunction in Takotsubo syndrome, without evidence of publication bias among the included studies.

Sensitivity analyses using leave-one-out exclusion procedures confirmed the robustness of the primary finding: across all iterations, the direction of effect remained unchanged, and the magnitude of the standardized mean difference consistently favored lower LVEF in TTS compared with anterior STEMI (SMD range −0.69 to −0.92; *p* value range 0.001–0.002), indicating that no single study disproportionately influenced the overall result.

### 3.6. Effect of Takotsubo Syndrome Versus Anterior STEMI on LV-GLS

Six studies [22,23,24,25,26,27] comparing LV-GLS between TTS and anterior STEMI were included in the meta-analysis. Because of the marked heterogeneity among studies (Q = 117.0, *p* < 0.001; I^2^ = 95.7%; τ^2^ = 1.43), a random-effects model was used to obtain a conservative pooled estimate.

Several individual studies reported significantly lower GLS values in TTS compared with anterior STEMI (e.g., median 9.1 [3.5–13] vs. 10.5 [6.3–13]%, *p* = 0.01), reflecting the greater degree of transient diffuse myocardial dysfunction typically observed in stress-induced cardiomyopathy. However, these findings represent within-study comparisons and therefore reflect local patient populations, imaging timing, and analytic conditions specific to each study.

**Figure 4:**
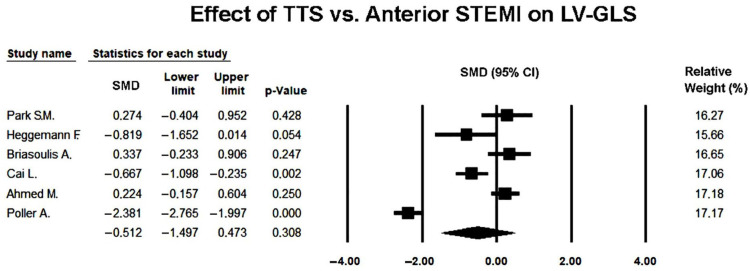
Forest plot illustrating the effect of TTS vs. anterior STEMI on LV-GLS [22,23,24,25,26,27]. LV-GLS, left ventricular global longitudinal strain; STEMI, ST-elevation myocardial infarction; TTS, takotsubo syndrome.

Under the random-effects model, the pooled SMD was −0.512 (95% CI −1.497 to 0.473; *p* = 0.308), indicating no statistically significant difference in global longitudinal strain between TTS and anterior STEMI during the acute phase (Figure 4).

This discrepancy between individual study results and the pooled estimate highlighted the impact of substantial between-study heterogeneity, as GLS values differed widely across included cohorts.

While most studies reported numerically lower GLS values in TTS, the wide confidence intervals and substantial heterogeneity suggested notable variability in study populations, echocardiographic timing, and strain analysis software. Thus, although the direction of effect tended to favor more impaired GLS in TTS, the meta-analytic evidence did not support a statistically robust difference once variability across studies was accounted for.

**Figure 5:**
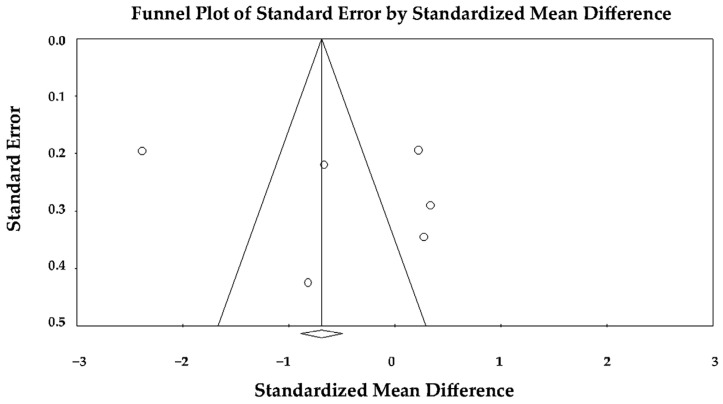
Begg’s funnel plot illustrating the assessment of publication bias in studies investigating the effect of TTS versus anterior STEMI on LV-GLS. LV-GLS, left ventricular global longitudinal strain; STEMI, ST-elevation myocardial infarction; TTS, takotsubo syndrome.

The funnel plot appeared slightly asymmetric (Figure 5), but Egger’s regression test did not indicate significant publication bias (intercept = 5.07 ± 7.88; t(4) = 0.64; two-tailed *p* = 0.55).

Therefore, the observed heterogeneity was unlikely to be explained by selective publication of positive findings. The forest plot showed that two studies [23,27] had the largest effect sizes, contributing disproportionately to between-study variability.

Meta-regression analysis incorporating demographic variables, software vendor, and echocardiographic acquisition timing did not identify any significant moderators of the GLS effect size. Neither mean age (coefficient 0.034, *p* = 0.839) nor the use of non-GE software platforms (coefficient 0.777, *p* = 0.446) demonstrated a statistically meaningful influence on the GLS difference between TTS and anterior STEMI. Importantly, STE timing—ranging from <24 h to up to 7 days after symptom onset—also did not emerge as a significant predictor (coefficient 0.034, *p* = 0.280), despite its plausible physiological relevance, particularly in TTS where early recovery of longitudinal strain may occur (Table 6).

Taken together, the overall test of moderators was not significant, indicating that these study-level characteristics—including imaging timing—did not explain the very high between-study heterogeneity observed across GLS analyses.

Overall, the random-effects analysis suggested that although TTS tends to show more impaired GLS compared with anterior STEMI, the difference was not statistically significant once heterogeneity was accounted for, and there was no evidence of publication bias across the included studies.

Sensitivity analyses using leave-one-out procedures demonstrated that the overall direction of effect was stable across all iterations, with SMDs ranging from −0.015 to −0.245 (*p* value range 0.49–0.96), confirming that no single study materially influenced the pooled estimate and that the non-significant difference in GLS between TTS and anterior STEMI was robust to the exclusion of individual studies.

### 3.7. Effect of Takotsubo Syndrome Versus Anterior STEMI on LV-ALS

Six studies [22,23,24,25,26,27] comparing ALS between TTS and anterior STEMI were included in the quantitative analysis. Owing to significant inter-study variability (Q = 72.98, *p* < 0.001; I^2^ = 93.1%; τ^2^ = 0.85), a random-effects model was applied.

**Figure 6:**
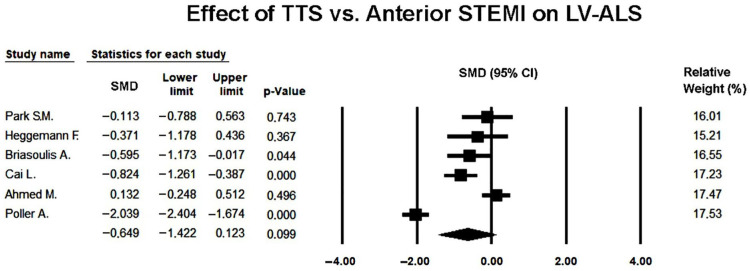
Forest plot showing the effect of TTS vs. anterior STEMI on LV-ALS [22,23,24,25,26,27]. LV-ALS, left ventricular apical longitudinal strain; STEMI, ST-elevation myocardial infarction; TTS, takotsubo syndrome.

The pooled SMD under the random-effects model was −0.649 (95% CI −1.422 to 0.123; *p* = 0.099), indicating a non-significant trend toward lower (more impaired) apical strain in TTS compared with anterior STEMI during the acute phase (Figure 6).

The large heterogeneity suggested that differences in imaging protocols, timing of echocardiography, and strain analysis software may have influenced the magnitude of observed effects.

Visual inspection of the forest plot revealed that most studies reported lower ALS values in TTS, consistent with greater apical dysfunction, although two studies [25,27] displayed larger effect sizes that contributed substantially to overall heterogeneity.

**Figure 7:**
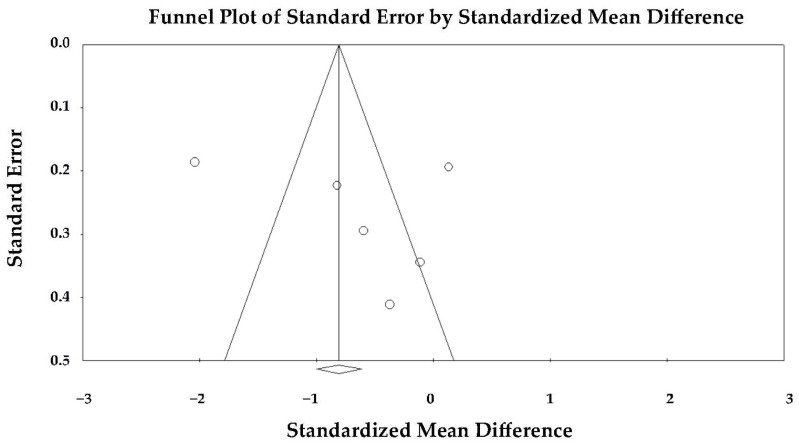
Begg’s funnel plot for the assessment of publication bias in studies evaluating the effect of TTS versus anterior STEMI on LV-ALS. LV-ALS, left ventricular apical longitudinal strain; STEMI, ST-elevation myocardial infarction; TTS, takotsubo syndrome.

The funnel plot appeared relatively symmetric (Figure 7), and Egger’s regression test did not reveal evidence of publication bias (intercept = 4.41 ± 6.02; t(4) = 0.73; two-tailed *p* = 0.50), suggesting that small-study effects were unlikely to have materially affected the results.

In summary, the random-effects meta-analysis indicated that patients with TTS tended to exhibit more pronounced impairment of apical longitudinal strain than those with anterior STEMI, but the difference did not reach statistical significance once heterogeneity was accounted for, and no publication bias was detected among the included studies.

Sensitivity analyses using a leave-one-out approach demonstrated that the direction of effect remained consistent across all iterations (SMD range −0.567 to −0.219; *p*-value range < 0.001–0.41, respectively), indicating a persistent but variably significant trend toward more impaired apical strain in TTS. No single study disproportionately altered the pooled estimate, confirming that the overall non-significant difference in ALS was robust to the exclusion of individual datasets.

### 3.8. Effect of Takotsubo Syndrome Versus Anterior STEMI on LV-MLS

Five studies [23,24,25,26,27] comparing MLS between TTS and anterior STEMI were included in the meta-analysis. Because of the marked heterogeneity among studies (Q = 141.7, *p* < 0.001; I^2^ = 97.2%; τ^2^ = 2.06), a random-effects model was used.

**Figure 8:**
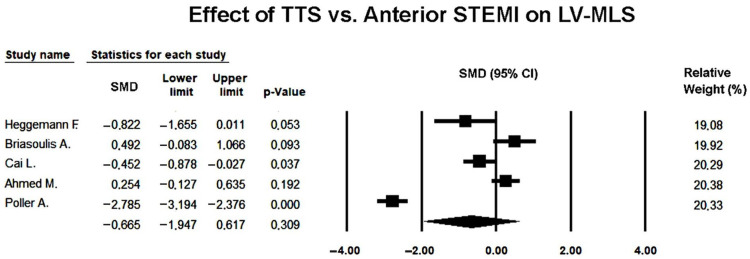
Forest plot illustrating the effect of TTS vs. anterior STEMI on LV-MLS [23,24,25,26,27]. LV-MLS, left ventricular mid-ventricular longitudinal strain; STEMI, ST-elevation myocardial infarction; TTS, takotsubo syndrome.

Under the random-effects model, the pooled SMD was −0.665 (95% CI −1.947 to 0.617; *p* = 0.309), indicating no statistically significant difference in mid-ventricular strain between TTS and anterior STEMI during the acute phase (Figure 8).

Although individual studies generally reported lower strain values in TTS, the high degree of heterogeneity suggested that methodological variability (differences in imaging timing, speckle-tracking software, and regional analysis criteria) influenced the consistency of results.

The forest plot demonstrated a predominance of studies favoring greater impairment in TTS, though the magnitude and direction of effects varied widely. Two studies [25,27] showed larger negative differences, thereby contributing substantially to the observed heterogeneity.

**Figure 9:**
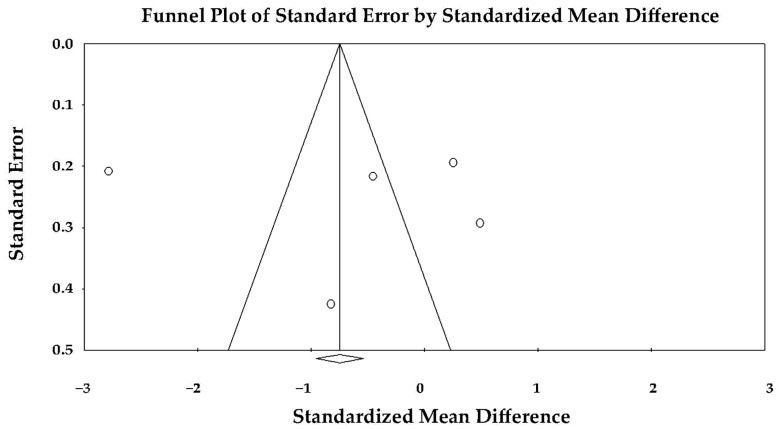
Begg’s funnel plot illustrating the assessment of publication bias in studies examining the effect of TTS versus anterior STEMI on LV-MLS. LV-MLS, left ventricular mid-ventricular longitudinal strain; STEMI, ST-elevation myocardial infarction; TTS, takotsubo syndrome.

Visual inspection of the funnel plot revealed no marked asymmetry (Figure 9), and Egger’s regression test confirmed the absence of publication bias (intercept = 2.66 ± 12.36; t(3) = 0.22; two-tailed *p* = 0.84). Therefore, the variability among studies was more likely attributable to methodological and population differences rather than selective reporting.

Overall, the random-effects analysis indicated that mid-ventricular strain tended to be more impaired in TTS compared with anterior STEMI, but this difference did not reach statistical significance once inter-study heterogeneity was accounted for, and no publication bias was detected.

Sensitivity analyses using a leave-one-out approach showed that the direction of effect remained stable across all iterations (SMD range −0.231 to −0.085; *p*-value range = 0.533–0.844), indicating a persistent but non-significant trend toward more impaired mid-ventricular strain in TTS. None of the exclusions materially altered the pooled estimate, confirming that the overall non-significant difference in MLS was robust to individual study removal.

### 3.9. Effect of Takotsubo Syndrome Versus Anterior STEMI on LV-BLS

Six studies [22,23,24,25,26,27] evaluating BLS in patients with TTS and anterior STEMI were included in the quantitative synthesis. Owing to substantial heterogeneity among studies (*Q* = 166.7, *p* < 0.001; I^2^ = 97.0%; τ^2^ = 2.13), a random-effects model was used.

**Figure 10:**
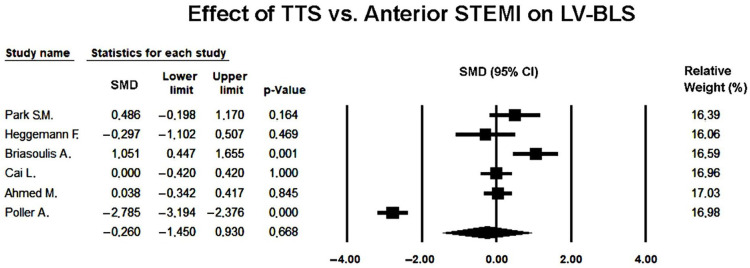
Forest plot illustrating the effect of TTS vs. anterior STEMI on LV-BLS [22,23,24,25,26,27]. LV-BLS, left ventricular basal longitudinal strain; STEMI, ST-elevation myocardial infarction; TTS, takotsubo syndrome.

The pooled SMD under the random-effects model was −0.260 (95% CI −1.450 to 0.930; *p* = 0.668), indicating no statistically significant difference in basal strain between TTS and anterior STEMI during the acute phase (Figure 10).

Although several studies showed numerically less impaired basal strain in TTS, the overall effect was inconsistent across studies and highly variable.

The forest plot demonstrated wide confidence intervals and opposing effect directions, reflecting pronounced heterogeneity. This variability was likely attributable to differences in echocardiographic timing, image quality, and analytic methodology among the included studies.

**Figure 11:**
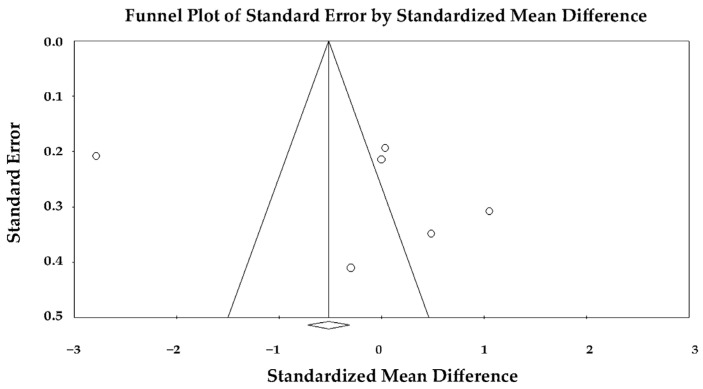
Begg’s funnel plot used for the assessment of publication bias in studies evaluating the effect of TTS versus anterior STEMI on LV-BLS. LV-BLS, left ventricular basal longitudinal strain; STEMI, ST-elevation myocardial infarction; TTS, takotsubo syndrome.

The funnel plot appeared symmetric (Figure 11), and Egger’s regression test did not indicate publication bias (intercept = 7.05 ± 9.41; *t*(4) = 0.75; two-tailed *p* = 0.50), suggesting that small-study effects were unlikely to have influenced the pooled result.

Overall, the random-effects meta-analysis indicated that basal longitudinal strain was not significantly different between TTS and anterior STEMI, and that no evidence of publication bias was detected. The findings highlighted the high degree of variability across studies and the need for standardized regional strain assessment protocols.

Sensitivity analyses using a leave-one-out approach demonstrated that the effect estimates remained consistently non-significant across all iterations (SMD range 0.124 to 0.451; *p*-value range = 0.234–0.751), indicating that no single study materially influenced the pooled result and that the overall non-significant difference in BLS between TTS and anterior STEMI was robust to the exclusion of individual datasets.

### 3.10. Effect of Takotsubo Syndrome Versus Anterior STEMI on LV-GRS

Two studies [23,27] comparing LV-GRS between TTS and anterior STEMI were included in the quantitative synthesis. Since the heterogeneity was negligible (*Q* = 0.76, *p* = 0.38; I^2^ = 0%), both fixed- and random-effects models yielded identical results.

**Figure 12:**
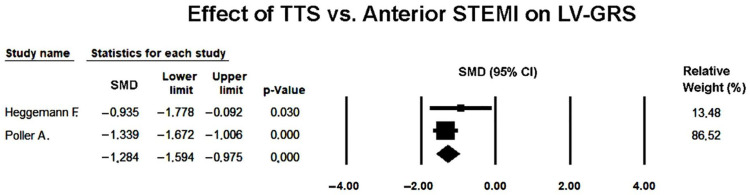
Forest plot showing the effect of TTS vs. anterior STEMI on LV-GRS [23,27]. LV-GRS, left ventricular global radial strain; STEMI, ST-elevation myocardial infarction; TTS, takotsubo syndrome.

The pooled SMD was −1.284 (95% CI −1.594 to −0.975; *p* < 0.001), indicating that GRS was significantly lower in TTS compared with anterior STEMI during the acute phase (Figure 12).

This finding reflected more severe impairment of radial contractility in TTS, consistent with the extensive circumferential involvement of myocardial segments typical of stress-induced cardiomyopathy.

The forest plot demonstrated a consistent direction of effect across both studies, with non-overlapping confidence intervals indicating strong agreement. The relative contribution of Poller et al. [27] was higher due to its larger sample size and smaller variance.

Because of the limited number of included studies, publication bias could not be formally assessed, but the lack of heterogeneity and consistency in the observed effects supported the robustness of the pooled estimate.

Overall, this analysis indicated that patients with TTS exhibited significantly lower LV global radial strain than those with anterior STEMI, suggesting greater global impairment of radial myocardial deformation in the acute phase of TTS.

### 3.11. Publication Bias Assessment

Inter-rater reliability for the risk-of-bias assessment, quantified using Cohen’s kappa coefficient, demonstrated substantial agreement between reviewers (κ = 0.80). Across the six included studies, overall methodological quality was predominantly fair, with one study rated as good according to the NIH Quality Assessment Tool for Case–Control Studies [19] (Supplementary Materials S3).

Although research objectives and case/control definitions were generally well described and imaging protocols were applied consistently, recurrent limitations included the absence of sample size justification, but more importantly, the lack of assessor blinding, which is particularly critical for speckle-tracking analysis given its susceptibility to subjective image selection and region-of-interest placement, as well as limited adjustment for confounding variables. These weaknesses affect internal validity but do not suggest systematic bias in effect direction.

**Figure 13:**
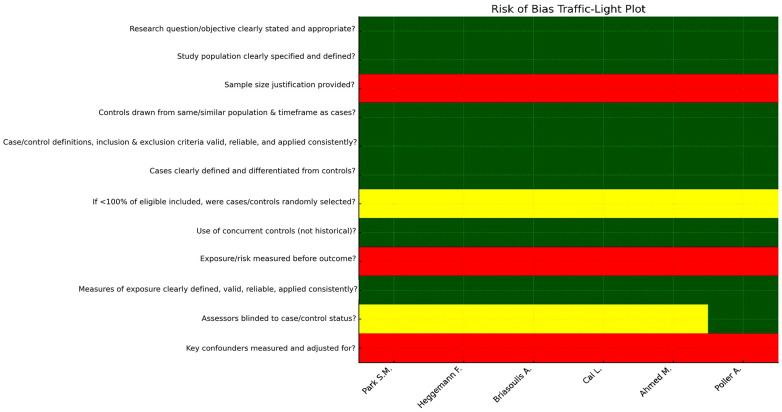
Traffic-light plot of risk of bias across included studies [22,23,24,25,26,27]. Traffic-light visualization summarizing the domain-specific risk-of-bias assessment for all six included studies, based on the NIH Quality Assessment Tool for Case–Control Studies [19]. Colors indicate the judgment for each domain (green = “YES”, yellow = “NR”, red = “NO”).

**Figure 14:**
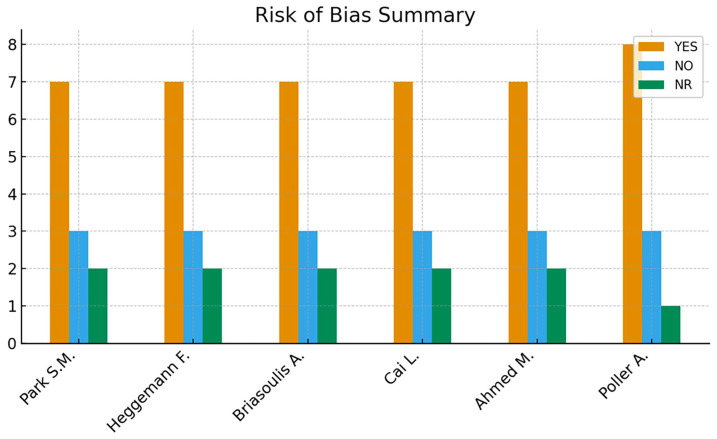
Summary bar chart of risk-of-bias judgments [22,23,24,25,26,27]. Summary bar chart representing the overall distribution of risk-of-bias ratings (“YES”, “NO”, “NR”) across the 12 NIH assessment domains for each study.

To provide a structured overview of methodological quality, a traffic-light plot summarizing domain-specific risk-of-bias judgments for each study (Figure 13) and a complementary summary bar chart quantifying the distribution of “YES”, “NO”, and “NR” assessments across domains were generated (Figure 14). These visualizations highlight recurrent methodological gaps—particularly absence of power calculations, lack of blinding, and unmeasured confounding—which were consistent across most studies.

In line with this, visual inspection of funnel plots and Egger’s regression testing revealed no significant small-study effects for GLS, ALS, MLS, or BLS, and no evidence of publication bias for LVEF. Overall, the heterogeneity observed across studies was more plausibly explained by methodological variation than by selective reporting.

## 4. Discussion

### 4.1. Main Findings

The present meta-analysis provides a comprehensive quantitative synthesis comparing LV mechanics and conventional echocardiographic characteristics between TTS and anterior STEMI due to LAD artery occlusion. While descriptive pooled data suggested significant differences in several longitudinal strain measures, the formal random-effects meta-analyses revealed that these differences did not reach statistical significance for GLS, ALS, MLS, or BLS once substantial inter-study heterogeneity was accounted for. Therefore, although numerical trends favored more impaired longitudinal deformation in TTS, the evidence supporting true differences in global or regional longitudinal strain is limited, heterogeneous, and should be interpreted as hypothesis-generating rather than definitive. In contrast, LVEF and GRS remained significantly lower in TTS in the pooled analyses, representing the most robust discriminative mechanical parameters identified in this study. Importantly, these findings must be viewed in light of the modest methodological quality of the included studies, the small sample sizes, and the recognized vendor- and operator-dependence of STE measurements; therefore, strain parameters cannot be considered standalone diagnostic discriminators at this stage but rather adjunctive tools to be interpreted cautiously alongside clinical and conventional echocardiographic findings.

Six observational studies, conducted across South Korea, Germany, the United States, and Sweden, were included, comprising 221 patients with TTS and 290 with anterior STEMI. All studies assessed LV function using 2D-STE in the acute phase, adhering to Mayo Clinic or ESC diagnostic criteria for TTS and angiographically confirmed LAD occlusion for STEMI. By pooling these data, our analysis provides an integrated comparison of global, regional, and radial deformation patterns between the two conditions.

Clinically, patients with TTS were older, predominantly female, and had a lower prevalence of traditional cardiovascular risk factors compared with those with anterior STEMI, consistent with the well-recognized epidemiological differences between TTS and ischemic myocardial infarction.

Hemodynamically, TTS was associated with higher admission heart rates and lower systolic blood pressure, consistent with an acute stress response rather than ischemic pump failure. Despite overlapping clinical presentation, ST-segment elevation was present in nearly all STEMI cases but only in about half of TTS patients. The magnitude of myocardial injury, reflected by peak troponin I levels, was profoundly lower in TTS, confirming the absence of significant myonecrosis in stress-induced cardiomyopathy. Moreover, physical or emotional stressors were identified as precipitating factors in over 75% of TTS cases, highlighting the psychosomatic dimension of the disorder. This constellation of clinical findings aligns with the concept that TTS represents a neurohormonal storm rather than a primary vascular occlusion.

From a conventional echocardiographic perspective, TTS patients demonstrated significantly lower LVEF compared with anterior STEMI, indicating more severe acute systolic dysfunction despite the absence of obstructive coronary disease. LV dimensions and volumes were largely comparable between groups, suggesting that the reduced ejection fraction in TTS reflects diffuse myocardial stunning rather than geometric remodeling. The interventricular septum was slightly thinner in TTS, consistent with the typically non-hypertrophic phenotype of these patients. Importantly, the observation of more depressed LVEF in TTS supports the usefulness of conventional transthoracic echocardiography as an early discriminative tool, helping clinicians suspect stress-induced cardiomyopathy even before advanced imaging is available.

Quantitative strain analysis provided deeper mechanistic insight. Across most individual studies, GLS tended to be lower in TTS compared with anterior STEMI, but the pooled meta-analytic estimate did not reach statistical significance. Similarly, ALS, MLS, and BLS showed a consistent numerical trend toward more impaired deformation in TTS, yet these findings should be viewed cautiously given the high heterogeneity and lack of statistical significance. In this context, the apical-to-basal gradient frequently described in individual TTS studies should be regarded as a descriptive clinical pattern rather than a confirmed result of the pooled meta-analytic evidence. Conversely, GRS was significantly lower in TTS, reflecting impaired radial thickening consistent with diffuse myocardial stunning. Taken together, these deformation patterns are suggestive of, but do not conclusively establish, broader longitudinal functional involvement in TTS compared with LAD-mediated STEMI; only GRS achieved statistical significance in pooled analysis.

Collectively, these multi-parametric findings are consistent with, but do not definitively demonstrate, a mechanical profile in TTS characterized by profound yet reversible global systolic impairment, with potential involvement of subendocardial longitudinal fibers, superimposed on relatively preserved geometry and disproportionately modest biomarker elevation. This contrasts with anterior STEMI, in which systolic impairment is regional, irreversible, and confined to the LAD territory. The integrated analysis of clinical, conventional echocardiographic, and deformation parameters underscores the unique pathophysiological substrate of TTS—a syndrome driven by transient catecholamine-mediated myocardial dysfunction rather than ischemic necrosis—and supports, albeit with appropriate caution regarding longitudinal strain, the incremental diagnostic value of strain imaging in differentiating the two conditions. Importantly, STE-derived abnormalities—particularly longitudinal strain patterns—should be used as adjunctive rather than standalone discriminators, given the non-significant pooled results and substantial heterogeneity.

### 4.2. Pathophysiological Mechanisms of LVEF and GLS Impairment in TTS vs. STEMI

Although TTS and anterior STEMI share similar acute LV systolic dysfunction, the underlying mechanisms of GLS impairment differ profoundly. In STEMI, GLS reduction primarily reflects irreversible ischemic necrosis within the LAD coronary artery territory, resulting in loss of contractile tissue, replacement fibrosis, and persistent mechanical dyssynchrony. Conversely, in TTS, GLS impairment occurs in the absence of obstructive coronary artery disease and is largely reversible, representing a functional rather than structural myocardial injury [4,8].

Catecholamine-mediated myocardial stunning remains the dominant hypothesis: excessive β-adrenergic stimulation during emotional or physical stress produces intracellular calcium overload, oxidative stress, and microvascular dysfunction, leading to transient myocyte injury and reduced contractile reserve [7,28].

Speckle-tracking echocardiography has suggested a characteristic apical-to-basal gradient of strain reduction in TTS, consistent with regional differences in sympathetic innervation density and β-adrenoceptor distribution [29]. The apical myocardium exhibits higher β_2_-receptor density and thinner wall-stress tolerance, predisposing it to greater catecholamine-induced stunning, while the basal segments—rich in β_1_-receptors—may develop compensatory hypercontractility [30]. The net effect is a “functional decoupling” between apical and basal mechanics, producing the classical ballooning pattern and depressed global strain values. However, because this gradient was not statistically confirmed in our pooled quantitative analysis and was limited by substantial heterogeneity, it should be regarded as a physiologically plausible and frequently reported pattern rather than a meta-analytically established finding. In contrast, GLS impairment in anterior STEMI is geographically confined to the LAD perfusion zone, with preserved strain in remote territories.

Importantly, the mechanisms governing LVEF impairment differ between the two entities. In TTS, LVEF is often more severely depressed because systolic dysfunction is diffuse and involves multiple contiguous myocardial regions beyond a single coronary territory. By contrast, in anterior STEMI, LVEF reduction reflects the extent of irreversible necrosis within the LAD territory, yet global pump function is partially preserved through compensatory hyperkinesia of non-ischemic or remote LV segments. This “recruitment” of viable myocardium—mediated by increased adrenergic drive, enhanced regional perfusion, and augmented contractile performance in territories unaffected by ischemia—can mitigate the reduction in overall stroke volume despite substantial regional akinesia [31,32]. Such compensatory remodeling, consistently demonstrated by imaging studies linking remote hyperkinesis with preserved perfusion, is well-described in acute infarction physiology and may explain why, in the present meta-analysis, LVEF was paradoxically less reduced in STEMI compared with the more globally affected TTS phenotype.

Microvascular dysfunction contributes differently to both entities. In STEMI, no-reflow phenomena and microembolization exacerbate infarct expansion and limit recovery despite revascularization. In TTS, reversible microcirculatory spasm and endothelial dysfunction lead to patchy, non-territorial hypoperfusion that parallels the distribution of strain abnormalities [33]; similarly, other reviews highlight direct catecholamine toxicity causing contraction-band necrosis and transient myocytolysis, explaining the disproportion between marked functional impairment and modest troponin elevation [34,35].

Growing mechanistic evidence suggests that TTS triggers a disproportionately pro-inflammatory immune response characterized by a predominance of classically activated M1 macrophages over alternatively activated M2 macrophages [36]. This imbalance promotes a more sustained and less reparative inflammatory environment compared with STEMI, where a coordinated transition from M1 to M2 phenotypes typically supports tissue healing and scar formation. A protracted M1-dominant response in TTS may contribute to diffuse myocardial stunning, delayed functional recovery, and the absence of an organized reparative phase, pointing to fundamental pathophysiological differences from STEMI.

The temporal dynamics of GLS recovery further distinguish TTS from STEMI. Serial imaging studies demonstrate rapid normalization of GLS within weeks in TTS, reflecting restoration of calcium handling and microvascular tone [37], whereas STEMI patients exhibit slower and often incomplete recovery due to irreversible tissue loss. Collectively, these findings suggest that GLS impairment in TTS arises from transient, diffuse myocardial stunning driven by catecholaminergic and microvascular mechanisms, whereas in STEMI it represents fixed regional loss of contractility from ischemic necrosis. Similarly, the mechanisms of LVEF impairment diverge: diffuse, transient contractile depression in TTS versus regional, partially compensated dysfunction in STEMI. The contrasting pathophysiology underscores why both syndromes may exhibit similar degrees of systolic dysfunction acutely but diverge profoundly in recovery potential and long-term prognosis. In this context, the nature of the triggering event emerges as a relevant modifier of myocardial response in TTS. In our pooled cohort, emotional triggers (36.8%) and physical triggers (41.4%) were similarly represented; however, recent evidence demonstrates that trigger patterns differ markedly according to age. Studies in octogenarians and nonagenarians show that physical stressors—such as acute illness, surgery, or frailty-related physiological perturbations—predominate overwhelmingly in older adults, whereas emotional triggers are more frequent in younger or middle-aged women [38,39,40]. Large registry data further confirm that advancing age is independently associated with a shift toward physical precipitants and a more severe acute clinical profile, including higher rates of cardiogenic shock and complications [41]. These observations suggest that the myocardial susceptibility to catecholamine surges in elderly patients is more often elicited by systemic physiologic stress rather than emotional stimuli, providing additional insight into the heterogeneous presentations and mechanical phenotype of TTS across the lifespan.

### 4.3. Implications for Clinical Practice

The integration of STE into the clinical assessment of acute LV dysfunction has major implications for the differential diagnosis and management of TTS and anterior STEMI. Although both conditions can present with comparable electrocardiographic and biomarker profiles, their therapeutic approaches and prognostic trajectories differ substantially. The present meta-analysis reinforces that TTS exhibits significantly lower LVEF and markedly reduced GRS compared with anterior STEMI, reflecting the diffuse nature of myocardial stunning in stress cardiomyopathy. Importantly, our results indicate that conventional TTE—particularly LVEF assessment—retains diagnostic value in the early evaluation of acute LV dysfunction. The observation that LVEF is often more profoundly reduced in TTS than in anterior STEMI, despite comparable clinical presentations, suggests that simple bedside echocardiography may aid the initial differentiation between the two entities before more advanced imaging becomes available.

Identifying descriptive patterns of GLS impairment can further assist clinicians in distinguishing TTS from ischemic injury at an early stage, particularly when coronary angiography is delayed or inconclusive. The typical apical-to-basal gradient of strain reduction, with relatively preserved basal contractility, is frequently reported in clinical practice and may support suspicion of TTS, but this feature was not statistically confirmed in our pooled analysis and should therefore be viewed as a supportive, non-definitive imaging clue rather than a validated diagnostic marker. Moreover, STE enables a regional, segment-by-segment analysis of myocardial deformation that conventional LVEF cannot provide. In the context of anterior STEMI, STE highlights the territorial distribution of injury confined to the LAD watershed, while in TTS it reveals diffuse involvement extending beyond a single vascular territory. This capacity for regional mapping of myocardial mechanics represents a crucial complement to conventional echocardiography and may enhance diagnostic accuracy when interpreted in conjunction with clinical findings and other imaging parameters.

Beyond diagnostic value, GLS quantification offers incremental prognostic information in both entities. In STEMI, impaired GLS is a validated predictor of adverse remodeling, arrhythmias, and heart failure [42], whereas in TTS, early GLS measurement may provide complementary prognostic insight, identifying patients at higher risk of cardiogenic shock, LV thrombus formation, or delayed recovery [43,44]. Incorporating STE into the acute diagnostic pathway therefore facilitates individualized monitoring and may optimize the timing of follow-up imaging [45]. In TTS, GLS normalization during recovery can confirm functional recovery and guide de-escalation of therapy, while persistent strain abnormalities may prompt investigation for alternative or concomitant cardiomyopathies.

Moreover, awareness of the distinct pathophysiological basis of GLS impairment has therapeutic relevance. Unlike STEMI, where myocardial necrosis demands prompt reperfusion and secondary prevention, TTS primarily requires hemodynamic stabilization, avoidance of excessive catecholamine exposure, and management of LV outflow obstruction. Understanding that strain abnormalities in TTS—including depressed GRS and a characteristic apex-to-base gradient—largely reflect reversible myocardial stunning rather than infarction may prevent unnecessary invasive procedures and allow more tailored supportive strategies.

Finally, as GLS becomes increasingly integrated into risk stratification models, standardized acquisition protocols, vendor-independent strain analysis, and systematic reporting of reproducibility metrics are essential to ensure consistency across centers. The use of GLS in differentiating, monitoring, and prognosticating TTS and STEMI underscores its emerging role as a core component of precision echocardiography in contemporary cardiology, while recognizing that the discriminative value of longitudinal strain patterns remains limited by current evidence and substantial heterogeneity.

### 4.4. Limitations of the Included Studies

Several methodological and technical limitations should be acknowledged when interpreting the findings of this meta-analysis. Most included studies were small, single-center investigations with retrospective or observational designs, which may introduce selection and reporting bias. Diagnostic criteria for TTS and anterior STEMI were not fully uniform across studies, and the timing of echocardiographic acquisition varied considerably—from within the first 24 h to several days after symptom onset—likely contributing to the substantial heterogeneity observed across all strain parameters (I^2^ often >90%). The absence of standardized timing also limits the ability to compare truly hyperacute with subacute imaging phases, which represent different physiological stages in both TTS and STEMI and may have influenced effect estimates.

From a technical standpoint, STE has recognized limitations. Inter-vendor variability remains substantial, as platforms such as GE EchoPAC, TomTec, and Philips QLAB use different proprietary algorithms for speckle tracking and strain computation, influencing absolute GLS values and regional strain patterns [46]. Image quality, frame-rate settings, and region-of-interest placement also introduce variability, and STE measurements are sensitive to loading conditions, such as preload and afterload fluctuations, which may transiently alter myocardial deformation [47]. Extrinsic mechanical factors—including chest wall deformities, hyperinflated lungs, or suboptimal acoustic windows—may further impair tracking accuracy and reproducibility [48]. Notably, few studies reported intra- or inter-observer reproducibility metrics, limiting assessment of measurement reliability. Taken together, these factors underscore that strain measurements remain operator-dependent and technically variable, reducing confidence in effect estimates derived from small, heterogeneous datasets.

Several studies lacked blinded image interpretation and did not adjust for key confounders such as age, sex, cardiovascular risk factors, or hemodynamic status. The absence of such adjustments likely contributed to unexplained between-study heterogeneity, as confirmed by meta-regression analyses showing that standard clinical variables did not account for variability in LVEF or GLS effect sizes. Additionally, most studies did not provide longitudinal follow-up, preventing characterization of recovery patterns or long-term prognostic implications.

A further important limitation is that all included studies exclusively investigated apical variants of TTS. Consequently, the pooled strain patterns reported in this meta-analysis cannot be generalized to mid-ventricular, basal, or focal TTS variants, which together represent a non-negligible proportion of clinical TTS presentations and differ substantially in regional wall-motion abnormalities and deformation profiles. This restriction may have led to an overrepresentation of apical dysfunction and limits the external validity of our findings across the full phenotypic spectrum of TTS.

Another relevant limitation concerns the analysis of GRS. Although GRS was significantly lower in TTS compared with anterior STEMI, this conclusion is based on only two studies [23,27], including 69 TTS and 158 STEMI patients overall, one of which [23] contributed merely 24 participants. This limited evidence base reduces the robustness of the pooled estimate and cautions against overinterpreting the clinical applicability of GRS as a discriminatory parameter. Consequently, these findings should be regarded as preliminary and warrant confirmation in larger, prospective studies with standardized radial strain acquisition before being considered for routine diagnostic use.

Finally, although funnel plots and Egger’s testing did not indicate statistically significant publication bias for most parameters, the predominance of small observational studies means that selective publication of negative or neutral findings cannot be fully excluded. Overall, the combination of modest methodological quality, substantial heterogeneity, and known technical limitations of STE indicates that the strain findings of this meta-analysis should be interpreted with caution. These constraints further reinforce that STE-derived parameters cannot currently be recommended as standalone discriminators between TTS and STEMI and should instead be used as adjunctive tools pending validation in larger, standardized, prospective studies.

## 5. Conclusions

This meta-analysis suggests that TTS and anterior STEMI caused by LAD coronary occlusion present with comparable degrees of acute LV systolic dysfunction, yet arise from fundamentally distinct mechanical and pathophysiological processes. In TTS, the most robust pooled findings were the significantly lower LVEF and GRS, indicating diffuse, largely reversible impairment consistent with catecholamine-mediated myocardial stunning, whereas anterior STEMI reflects a focal, irreversible ischemic injury confined to the LAD perfusion territory.

Although longitudinal strain parameters showed only non-significant trends toward greater impairment in TTS once heterogeneity was accounted for, the descriptive pattern of apical-to-basal involvement remains clinically characteristic and may assist diagnostic reasoning when interpreted cautiously and in conjunction with other imaging and clinical features.

The consistently lower ejection fraction and reduced radial strain observed in TTS help define a deformation profile that is consistent with—but does not definitively establish—a distinction from ischemic cardiomyopathy despite similar initial presentations. These findings emphasize the diagnostic value of conventional transthoracic echocardiography and highlight 2D-STE as a useful tool for detecting the diffuse mechanical signature of stress-induced cardiomyopathy. However, given the small sample sizes, methodological limitations, and technical variability of strain analysis, STE-derived parameters should not be considered standalone discriminators between TTS and anterior STEMI but rather adjunctive elements within a broader multimodal assessment.

Together, these results support the value of integrating 2D-STE into the diagnostic workflow for patients presenting with acute LV dysfunction and suggest its potential utility in distinguishing TTS from LAD-related anterior STEMI, while emphasizing that confirmation in larger, standardized prospective studies is needed before firm diagnostic thresholds can be established.

## Figures and Tables

**Table 1:** Summary of studies [22,23,24,25,26,27] comparing TTS effect on LV mechanics vs. anterior STEMI.

Study Name, Year and Country	Design	Size (n)	Mean Age (yrs)	Females (%)	STE Software	Main Findings in TTS vs. Anterior STEMI
Park S.M. (2009), S. Korea [22]	Prosp.	TTS = 13 STEMI = 24	TTS 70 STEMI = 67	TTS = 92 STEMI = 33	GE EchoPAC (Vivid 7)	↓ LVEF; ↓ GLS; ↓ ALS; ↓ MLS; ↓ BLS
Heggemann F. (2011), Germany [23]	Prosp.	TTS = 12 STEMI = 12	TTS = 72 STEMI = 64	TTS = 83 STEMI = 25	TomTec 2D CPA	↓ LVEF; ↓ GLS; ↓ ALS; ↓ MLS; ↓ BLS; ↓ GRS
Briasoulis A. (2016), USA [24]	Retrosp.	TTS = 24 STEMI = 24	TTS = 68 STEMI = 66	TTS = 96 STEMI = 41	Philips QLAB (iE33)	↔ LVEF; ↔ GLS; ↑ BLS; ↔ ALS; ↔ MLS
Cai L. (2017), USA [25]	Retrosp.	TTS = 55 STEMI = 36	TTS = 69 STEMI = 64	TTS = 88 STEMI = 38	GE EchoPAC (Vivid E9)	↔ LVEF; ↔ GLS; ↓ ALS; ↓ MLS; ↔ BLS
Ahmed M. (2020), USA [26]	Retrosp.	TTS = 60 STEMI = 48	TTS = 70 STEMI = 64	TTS = 89 STEMI = 42	GE EchoPAC (Vivid E9)	↔ LVEF; ↔ GLS; ↑ ALS/MLS; ↓ BLS
Poller A. (2025), Sweden [27]	Prosp.	TTS = 57 STEMI = 146	TTS = 71 STEMI = 63	TTS = 91 STEMI = 39	TomTec Image Arena	↓ LVEF; ↔ GLS; ↔ ALS; ↔ MLS; ↔ BLS; ↓ GRS

ALS, apical longitudinal strain; BLS; basal longitudinal strain; GE, General Electric; GLS, global longitudinal strain; GRS, global radial strain; LV, left ventricular; LVEF, left ventricular ejection fraction; MLS, mid-ventricular longitudinal strain; Prosp., prospective; Retrosp., retrospective; STE, speckle tracking echocardiography; STEMI, ST-elevation myocardial infarction; TTS, takotsubo syndrome. ↓ = significantly more impaired in TTS vs. anterior STEMI; ↑ = significantly less impaired in TTS vs. anterior STEMI; ↔ = not statistically different in TTS vs. anterior STEMI.

**Table 2:** Clinical characteristics of TTS and anterior STEMI cohorts assessed by the include studies.

	TTS (n = 221)	Anterior STEMI (n = 290)	*p* Value
**Demographics**			
Age (yrs)	68.8 (62.9–73)	63.1 (58–66.9)	<0.001
Females (%)	91.4 (82–100)	49.2 (28.1–100)	<0.001
**Cardiovascular risk factors**			
Hypertension (%)	52.8 (23–72)	43.3 (33–58)	0.04
Diabetes (%)	10.9 (0–23)	20.8 (12.3–29)	0.004
Hypercholesterolemia (%)	21.8 (17.5–25)	41.4 (15.1–67)	<0.001
Smokers (%)	18 (14–23)	29.9 (21–42)	0.003
**Hemodynamics**			
Heart rate (bpm)	87.8 (82–94.3)	83.1 (79.9–89.6)	0.002
SBP (mmHg)	120.4 (98–132.3)	129.1 (119–136.1)	0.003
**ECG findings**			
ST elevation (%)	48 (32–77)	98 (92–100)	<0.001
**Blood tests**			
Peak troponin I (µg/L)	2.9 (2.3–3.3)	62.2 (29–93.7)	<0.001
**TTS trigger**			
Physical stress (%)	41.4 (28.1–54)	/	/
Emotional stress (%)	36.8 (17–47.3)	/	/

Data are presented as study-level summaries (medians with ranges). SBP, systolic blood pressure; STEMI, ST-elevation myocardial infarction; TTS, takotsubo syndrome.

**Table 3:** Conventional echocardiographic parameters measured by the included studies in TTS and anterior STEMI groups.

Conventional Echocardiographic Parameters	Number of Studies for Parameters Assessed (n° pts in TTS vs. STEMI)	TTS	Anterior STEMI	*p* Value
**IVS thickness (mm)**	3 (94 vs. 194)	9.4 (8.9–10.2)	10 (9.5–10.3)	0.03
**LV–EDD (mm)**	3 (141 vs. 218)	46.8 (45–49.1)	47.1 (45–50.7)	0.44
**LV–ESD (mm)**	3 (141 vs. 218)	33.3 (29–35.6)	34.2 (32–36.3)	0.29
**LV–EDVi (mL/m^2^)**	3 (130 vs. 218)	62.5 (49.5–80)	62.7 (45.2–90)	0.96
**LV–ESVi (mL/m^2^)**	3 (130 vs. 218)	36.9 (29.6–43)	35.2 (26.2–51)	0.61
**LVEF (%)**	6 (221 vs. 290)	34.2 (25.4–39.7)	41.6 (33.1–48.7)	0.03
**E/A ratio**	2 (70 vs. 170)	1.0 (0.7–1.3)	1.15 (1–1.3)	0.07
**E/e′ ratio**	3 (94 vs. 194)	12.2 (11.4–13)	12.3 (10.3–16.3)	0.88
**LAVi (mL/m^2^)**	2 (70 vs. 170)	30.8 (30.7–30.9)	31.4 (31.2–31.7)	0.05
**RVIT (mm)**	1 (55 vs. 36)	37 (37–37)	36 (36–36)	0.41
**TAPSE (mm)**	1 (55 vs. 36)	15 (15–15)	20 (20–20)	0.004

Data are presented as study-level summaries (medians with ranges). EDD, end-diastolic diameter; EDVi, end-diastolic volume indexed; ESD, end-systolic diameter; ESVi, end-systolic volume indexed; IVS, interventricular septum; LAVi, left atrial volume indexed; LV, left ventricular; LVEF, left ventricular ejection fraction; RVIT, right ventricular inflow tract; STEMI, ST-elevation myocardial infarction; TAPSE, tricuspid annular plane systolic excursion; TTS, takotsubo syndrome.

**Table 4:** STE-derived myocardial strain parameters obtained by the included studies in TTS vs. anterior STEMI cohorts.

STE Variables	Number of Studies for Parameters Assessed (n° pts in TTS vs. STEMI)	TTS	Anterior STEMI	*p* Value
**LV–GLS (%)**	6 (221 vs. 290)	−9.1 (−3.5, −13)	−10.5 (−6.3, −13)	0.01
**LV–ALS (%)**	6 (221 vs. 290)	−4.4 (−0.5, −9.8)	−6.4 (−3.4, −10)	0.001
**LV–MLS (%)**	5 (208 vs. 266)	−9.9 (−5.8, −13.4)	−11.6 (−5.7, −17.4)	0.006
**LV–BLS (%)**	6 (221 vs. 290)	−14.2 (−11.4, −18.1)	−14.2 (−7.3, −17.4)	0.03
**LV–GRS (%)**	2 (69 vs. 158)	15.7 (13.9, 17.5)	26.4 (23.8, 29.1)	<0.001

Data are presented as study-level summaries (medians with ranges). ALS, apical longitudinal strain; BLS, basal longitudinal strain; GLS, global longitudinal strain; GRS, global radial strain; LV, left ventricular; MLS, mid-ventricular longitudinal strain; STE, speckle tracking echocardiography; STEMI, ST-elevation myocardial infarction; TTS, takotsubo syndrome.

**Table 5:** Meta-regression analysis performed to evaluate whether cardiovascular risk factors such as hypertension, diabetes, or age contributed to variability in LVEF effect sizes.

Covariate	Coefficient	Standard Error	95% Lower	95% Upper	*p*-Value
Intercept	0.430	14.01	−27.02	27.883	0.975
Mean age TTS (yrs)	−0.086	0.191	−0.461	0.288	0.651
%Hypertension TTS	0.055	0.051	−0.046	0.155	0.286
%Diabetes TTS	0.133	0.120	−0.103	0.369	0.269

LVEF, left ventricular ejection fraction; TTS, takotsubo syndrome.

**Table 6:** Meta-regression assessing the influence of demographic and methodological variables—including age, STE software vendor, and timing of echocardiography—on GLS effect size.

Covariate	Coefficient	Standard Error	95% Lower	95% Upper	*p*-Value
Intercept	−4.228	11.81	−27.38	18.92	0.720
Mean age TTS (yrs)	0.034	0.165	−0.291	0.358	0.839
Software: nonGE	0.777	1.018	−1.210	2.773	0.446
STE timing (hours)	0.034	0.031	−0.027	0.094	0.280

GE, General Electric; GLS, global longitudinal strain; STE, speckle tracking echocardiography.

## Data Availability

Data extracted from included studies will be publicly available on Zenodo (https://zenodo.org).

## Supplementary Materials

The following supporting information can be downloaded at: https://www.mdpi.com/article/10.3390/jcm14248748/s1, Supplementary Materials S1: PRISMA 2020 checklist; Supplementary Materials S2: The full INPLASY record [49]; Supplementary Materials S3: NIH Quality Assessment of included studies [22,23,24,25,26,27].
