# Supplementary material for: Comparative Meta-Analysis of Left Ventricular Mechanics in Takotsubo Syndrome and Anterior STEMI Due to Left Anterior Descending Artery Occlusion
Source: J Clin Med. 2025 Dec 10;14(24):8748. doi: 10.3390/jcm14248748 (PMC12733908; doi:10.3390/jcm14248748)
Supplement: Supplementary file 1 [file jcm-14-08748-s001.zip › Supplementary Material S2.pdf]

International Platform of Registered Systematic Review and Meta-analysis Protocols

INPLASY

Comparative Meta-Analysis of Left Ventricular Mechanics in Takotsubo Syndrome and Anterior STEMI due to LAD Occlusion

INPLASY2025110021
doi: 10.37766/inplasy2025.11.0021
Received: 9 November 2025

Published: 9 November 2025

Sonaglioni, A; Nicolosi, GL; Baravelli, M.

Corresponding author:
Andrea Sonaglioni

sonaglioniandrea@gmail.com

Author Affiliation:
MultiMedica.

ADMINISTRATIVE INFORMATION

Support - No funding.

Review Stage at time of this submission - Preliminary searches.

Conflicts of interest - None declared.

INPLASY registration number: INPLASY2025110021

Amendments - This protocol was registered with the International Platform of Registered Systematic Review and Meta-Analysis Protocols (INPLASY) on 9 November 2025 and was last updated on 9 November 2025.

INTRODUCTION

Review question / Objective The aim of the present study is to perform a systematic review and meta-analysis of comparative studies evaluating left ventricular mechanics—particularly global longitudinal strain (GLS), regional longitudinal strain (apical, mid, basal), and left ventricular ejection fraction (LVEF)—in patients with Takotsubo syndrome versus those with anterior ST-elevation myocardial infarction (STEMI) due to left anterior descending (LAD) artery occlusion.

Rationale Advances in echocardiographic imaging, particularly two-dimensional speckle-tracking strain analysis, have enabled a more detailed assessment of left ventricular (LV) systolic mechanics beyond conventional ejection fraction (EF). Global longitudinal strain (GLS) has emerged as a sensitive marker of systolic function across a range of cardiac conditions, including acute coronary syndromes and chronic ischemic heart disease.

In the context of ST-elevation myocardial infarction (STEMI), speckle-tracking–derived longitudinal strain has been shown to predict infarct size, microvascular obstruction and subsequent LV remodeling, particularly in first anterior infarctions.
In Takotsubo syndrome (TTS), strain imaging offers unique insights into the pattern and extent of myocardial dysfunction. Recent literature and expert reviews describe a characteristic apical-to-basal gradient of longitudinal deformation, with severely impaired apical strain and relatively preserved or even hypercontractile basal segments, as well as potential differences in recovery patterns compared with ischemic injury.
Nevertheless, individual studies comparing LV mechanics between TTS and anterior STEMI are limited by relatively small sample sizes, single-center designs and methodological heterogeneity in echocardiographic acquisition and strain analysis. The true magnitude and regional distribution of LV mechanical impairment in TTS relative to left anterior descending (LAD) artery-related anterior STEMI therefore remain uncertain.

Sonaglioni et al. INPLASY protocol 2025110021. doi:10.37766/inplasy2025.11.0021 Downloaded from https://inplasy.com/inplasy-2025-11-0021/

Condition being studied Takotsubo syndrome (TTS), also known as stress-induced cardiomyopathy, is an acute, transient disorder of left ventricular (LV) function characterized by regional wall motion abnormalities extending beyond a single coronary artery territory, in the absence of obstructive coronary artery disease. It has been increasingly recognized over the past three decades as a distinct clinical entity predominantly affecting postmenopausal women and typically triggered by emotional or physical stress.

Large contemporary registries have confirmed that TTS accounts for a non-negligible proportion of patients initially presenting with suspected acute coronary syndrome, with a marked female predominance and a wide spectrum of clinical triggers and outcomes.

Comprehensive expert panel reviews have summarized the evolving concepts in epidemiology, diagnostic criteria, and management, highlighting TTS as a heterogeneous syndrome rather than a single disease entity.

Clinically, TTS often mimics an acute anterior ST-elevation myocardial infarction (STEMI), presenting with chest pain, ST-segment elevation, and elevation of cardiac biomarkers. This resemblance makes the differential diagnosis between TTS and true anterior STEMI particularly challenging in the acute phase. Despite similar initial presentations, the underlying pathophysiology differs substantially. TTS is thought to represent a form of myocardial stunning related to catecholamine excess and stress-related neurohumoral activation, whereas anterior STEMI is caused by prolonged ischemia due to an occlusive thrombus in the left anterior descending (LAD) artery.

Several mechanistic studies and reviews have proposed that TTS involves complex interactions between catecholamine toxicity, microvascular dysfunction, and myocardial energy metabolism, leading to transient but sometimes extensive LV dysfunction. In contrast, LAD-related anterior STEMI results in irreversible necrosis within a more clearly demarcated perfusion territory, followed by scar formation and chronic remodeling.

METHODS

Search strategy Two independent reviewers (A.S. and M.B.) systematically searched PubMed, Embase, and Scopus through October 2025 for all comparative studies evaluating LV function and mechanics by two-dimensional speckle-tracking echocardiography (2D-STE) in patients with TTS and anterior STEMI due to LAD occlusion. The following keywords and Boolean operators were used: “Takotsubo cardiomyopathy” OR “stress cardiomyopathy” OR “broken heart syndrome” OR “Takotsubo syndrome” AND “ST-elevation myocardial infarction” OR “anterior STEMI” OR “LAD occlusion” AND “echocardiography” OR “speckle tracking echocardiography” OR “strain imaging” OR “left ventricular strain” OR “global longitudinal strain” OR “LV mechanics.” No language or temporal restrictions were applied. Reference lists of included papers and relevant reviews were manually searched to identify additional eligible studies. Disagreements between reviewers were resolved by discussion or, when necessary, by consulting a third investigator.

Participant or population Studies were included if they met the following criteria: (1) case-control design directly comparing patients with TTS and anterior STEMI due to LAD occlusion; (2) quantitative assessment of LV function using 2D-STE; and (3) available data for at least one of the following parameters in both groups: GLS, regional longitudinal strain (apical, mid-ventricular, or basal), or LVEF. Exclusion criteria were: (1) studies including mixed cardiomyopathies or other causes of transient LV dysfunction (e.g., myocarditis or sepsis-induced cardiomyopathy); (2) absence of a comparison group with angiographically confirmed LAD occlusion; (3) lack of quantitative LV strain measurements; (4) imaging methods other than echocardiography (e.g., cardiac MRI strain only); and (5) conference abstracts, editorials, case reports, or narrative reviews without original data.

Intervention N/A.

Comparator Studies were included if they met the following criteria: (1) case-control design directly comparing patients with TTS and anterior STEMI due to LAD occlusion; (2) quantitative assessment of LV function using 2D-STE; and (3) available data for at least one of the following parameters in both groups: GLS, regional longitudinal strain (apical, mid-ventricular, or basal), or LVEF. Exclusion criteria were: (1) studies including mixed cardiomyopathies or other causes of transient LV dysfunction (e.g., myocarditis or sepsis-induced cardiomyopathy); (2) absence of a comparison group with angiographically confirmed LAD occlusion; (3) lack of quantitative LV strain measurements; (4) imaging methods other than echocardiography (e.g., cardiac MRI strain only); and (5) conference abstracts, editorials, case reports, or narrative reviews without original data.

Study designs to be included Case-control studies.

Sonaglioni et al. INPLASY protocol 2025110021. doi:10.37766/inplasy2025.11.0021 Downloaded from https://inplasy.com/inplasy-2025-11-0021/

Eligibility criteria Studies were included if they met the following criteria: (1) case-control design directly comparing patients with TTS and anterior STEMI due to LAD occlusion; (2) quantitative assessment of LV function using 2D-STE; and (3) available data for at least one of the following parameters in both groups: GLS, regional longitudinal strain (apical, mid-ventricular, or basal), or LVEF. Exclusion criteria were: (1) studies including mixed cardiomyopathies or other causes of transient LV dysfunction (e.g., myocarditis or sepsis-induced cardiomyopathy); (2) absence of a comparison group with angiographically confirmed LAD occlusion; (3) lack of quantitative LV strain measurements; (4) imaging methods other than echocardiography (e.g., cardiac MRI strain only); and (5) conference abstracts, editorials, case reports, or narrative reviews without original data.

Information sources Two reviewers (A.S. and M.B.) independently screened all titles and abstracts, followed by a full-text assessment of potentially eligible studies. Data were extracted from each study using a standardized collection form that included the following: (1) study characteristics (first author, year of publication, country, and study design); (2) population details (sample size, mean age, and proportion of female participants); (3) imaging protocol (vendor and software used for STE analysis, and timing of echocardiographic assessment—acute phase, recovery, or both); (4) conventional transthoracic echocardiography parameters (chamber dimensions and indices of LV systolic and diastolic function); (5) STE-derived LV functional parameters (GLS, apical, mid-ventricular, and basal longitudinal strain, and global radial strain when available); and (6) summary statistics (mean ± SD or median and interquartile range, together with reported p-values or confidence intervals). When results were available only in graphical form, numerical data were extracted using digital measurement software. Any discrepancies between reviewers were resolved through discussion and consensus.

Main outcome(s) To evaluate left ventricular mechanics—particularly global longitudinal strain (GLS), regional longitudinal strain (apical, mid, basal), and left ventricular ejection fraction (LVEF) —in patients with Takotsubo syndrome versus those with anterior STEMI due to LAD occlusion.

Quality assessment / Risk of bias analysis The methodological quality and risk of bias were independently evaluated by two reviewers (A.S. and G.L.N.) using the National Institutes of Health (NIH) Quality Assessment Tool for Case-Control Studies. Each study was classified as "good," "fair," or "poor" according to the NIH scoring criteria. Inter-rater reliability was assessed using Cohen's kappa coefficient (κ), and any discrepancies were resolved through discussion and consensus.

Strategy of data synthesis Continuous variables were reported as mean ± standard deviation (SD). For each echocardiographic parameter (GLS, apical, mid-ventricular, and basal longitudinal strain, global radial strain, and LVEF), standardized mean differences (SMDs) between TTS and anterior STEMI groups were calculated. Pooled estimates were obtained using a random-effects model (DerSimonian–Laird method) to account for anticipated heterogeneity among studies. Between-study heterogeneity was quantified using the I^2 statistic, with values of 25%, 50%, and 75% indicating low, moderate, and high heterogeneity, respectively. Publication bias was assessed both visually, through Begg's funnel plots, and statistically, using Egger's regression test. When sufficient data were available, meta-regression analyses were performed to evaluate the potential influence of covariates such as mean age, sex distribution, and the echocardiographic software vendor on GLS outcomes. Sensitivity analyses were conducted by sequentially excluding individual studies to assess the robustness of the pooled results. All statistical analyses were performed using Comprehensive Meta-Analysis software (version 3.0, Biostat, Englewood, NJ, USA). A two-tailed p-value < 0.05 was considered statistically significant.

Subgroup analysis For each echocardiographic parameter (GLS, apical, mid-ventricular, and basal longitudinal strain, global radial strain, and LVEF), standardized mean differences (SMDs) between TTS and anterior STEMI groups were calculated.

Sensitivity analysis Sensitivity analyses were conducted by sequentially excluding individual studies to assess the robustness of the pooled results.

Country(ies) involved Italy.

Keywords Takotsubo syndrome; stress cardiomyopathy; STEMI; LV mechanics; ventricular strain.

Contributions of each author

Author 1 - Andrea Sonaglioni - Author 1 drafted the manuscript.
Email: sonaglioniandrea@gmail.com

Sonaglioni et al. INPLASY protocol 2025110021. doi:10.37766/inplasy2025.11.0021 Downloaded from https://inplasy.com/inplasy-2025-11-0021/

Author 2 - Gian Luigi Nicolosi - Revision of the original manuscript.
Email: gianluigi.nicolosi@gmail.com
Author 3 - Massimo Baravelli - Supervision.
Email: massimo.baravelli@multimedica.it

Sonaglioni et al. INPLASY protocol 2025110021. doi:10.37766/inplasy2025.11.0021 Downloaded from https://inplasy.com/inplasy-2025-11-0021/
