# Supplementary material for: Comparative Meta-Analysis of Left Ventricular Mechanics in Takotsubo Syndrome and Anterior STEMI Due to Left Anterior Descending Artery Occlusion
Source: J Clin Med. 2025 Dec 10;14(24):8748. doi: 10.3390/jcm14248748 (PMC12733908; doi:10.3390/jcm14248748)
Supplement: Supplementary file 1 [file jcm-14-08748-s001.zip › Supplementary Material S3.pdf]

Criterion (NIH Case–Control Tool)	Park S.M. et al. [22]	Heggemann F. et al. [23]	Briasoulis A. et al. [24]	Cai L. et al. [25]	Ahmed M. et al. [26]	Poller A. et al. [27]
1. Research question/objective clearly stated and appropriate?	YES	YES	YES	YES	YES	YES
2. Study population clearly specified and defined?	YES	YES	YES	YES	YES	YES
3. Sample size justification provided?	NO	NO	NO	NO	NO	NO
4. Controls drawn from same/similar population & timeframe as cases?	YES	YES	YES	YES	YES	YES
5. Case/control definitions, inclusion & exclusion criteria valid, reliable, and applied consistently?	YES	YES	YES	YES	YES	YES
6. Cases clearly defined and differentiated from controls?	YES	YES	YES	YES	YES	YES
7. If <100% of eligible included, were cases/controls randomly selected?	NR	NR	NR	NR	NR	NR
8. Use of concurrent controls (not historical)?	YES	YES	YES	YES	YES	YES
9. Exposure/risk measured before outcome?	NO	NO	NO	NO	NO	NO
10. Measures of "exposure" (group status/echo variables) clearly defined, valid, reliable, and applied consistently?	YES	YES	YES	YES	YES	YES
11. Assessors of exposure/risk blinded to case/control status?	NR	NR	NR	NR	NR	YES
12. Key confounders measured and adjusted for (or matching accounted for) in analysis?	NO	NO	NO	NO	NO	NO
